# Processability of Atypical WC-Co Composite Feedstock by Laser Powder-Bed Fusion

**DOI:** 10.3390/ma13010050

**Published:** 2019-12-20

**Authors:** Mohaimen Al-Thamir, D. Graham McCartney, Marco Simonelli, Richard Hague, Adam Clare

**Affiliations:** 1 Centre for Additive Manufacturing, Faculty of Engineering, University of Nottingham, Nottingham NG 7 2RD, UK; eaxma20@exmail.nottingham.ac.uk (M.A.-T.); ezzms2@exmail.nottingham.ac.uk (M.S.); ezzrjh@exmail.nottingham.ac.uk (R.H.); 2 Advanced Component Engineering Laboratory (ACEL), University of Nottingham, Nottingham NG7 2RD, UK; 3 College of Engineering, University of Thi-Qar, 64001 Nasiriyah, Iraq; 4 Advanced Manufacturing, Faculty of Engineering, University of Nottingham, Nottingham NG7 2RD, UK; ezadm6@exmail.nottingham.ac.uk

**Keywords:** laser-powder bed fusion (L-PBF), WC_M_-Co, satelliting, single tracks, melt mode

## Abstract

Processing of tool materials for cutting applications presents challenges in additive manufacturing (AM). Processes must be carefully managed in order to promote the formation of favourable high-integrity ‘builds’. In this study, for the first time, a satelliting process is used to prepare a WC_M_-Co (12 wt.% Co) composite. Melting trials were undertaken to evaluate the consolidation behaviour of single tracks within a single layer. Tracks with continuous and relatively uniform surface morphology were obtained. These features are essential for high-quality AM builds in order to encourage good bonding between subsequent tracks within a layer which may reduce porosity within a 3D deposition. This study elucidates the formation of track irregularities, melting modes, crack sensitivity, and balling as a function of laser scanning speed and provides guidelines for future production of WC_M_-Co by laser powder-bed fusion.

## 1. Introduction

The fabrication of small customized cutting tool inserts from cemented carbides is one application area that could benefit from the adoption of additive manufacturing (AM) technology. The manufacture of carbide-rich special tools is a multistage process that is time consuming and costly. In particular, the design restrictions imposed by conventional production limit the integration of complex geometries containing desirable cooling channels, chip flute topology, and other functional features [1]. Specifically, laser powder-bed fusion (L-PBF) could replace the complex and expensive powder metallurgy manufacturing process that involves mixing, pressing, sintering and machining for such components [2].

Cemented carbides (termed cermets) based on tungsten carbide (WC) and cobalt (Co) are some of the most widely used cutting tool materials because of their outstanding combination of hardness and toughness compared to other tool materials, such as diamond or high-speed steels [3]. The main constituents in a WC cermet are a hexagonal WC phase and a Co binder phase. The WC exhibits a high hardness and melting point along with good electrical and thermal conductivity. The Co binder provides good toughness and ductility. The properties of the cermet can be adjusted by varying the ratio of the two components which are typically in the range 7–20 wt.% Co.

In recent years there has been extensive research on the processing of iron-, nickel-, titanium- and aluminium-based alloys amongst others by L-PBF [4,5,6,7]. The vast majority of this work has involved the use of pre-alloyed gas-atomised powder as feedstock material. There has been some research on alloy modification through the incorporation of a small fraction of additions either by powder blending or by a process known as “satelliting” but rarely have the additions exceeded a few volume percent [8]. If L-PBF is to be successfully deployed to manufacture tool materials it will be necessary to operate the process with a two-phase feedstock material in which the constituents have very different thermophysical properties eg WC and Co with around 10 to 20 vol.% Co.

There have been only a small number of studies on L-PBF of the WC-Co system. Uhlmann et al. [9] investigated agglomerated and pre-sintered WC-17 wt.% Co powder and found that there are two main contrary behaviours of the processed materials; brittle and dense materials were obtained in case of high energy densities, while high cobalt content and substantial porosity characteristics were presented in cases of low applied energy density. A similar trend in results has been confirmed by Schwanekamp, et al. [10] when agglomerated and sintered WC-12 wt.% Co powder, using different size ranges in the limit of (10–45) µm, have been examined.

The effect of chemical composition on resultant material porosity has been investigated by Fortunato et al. [11] using two kinds of atomised WC-Co powder in a size range of 15–45 µm. Although hot isostatic pressing was carried out after laser processing, three types of pores were observed in the material fabricated from a WC-11 wt.% Co powder; A (<10 µm), B (10–25 µm), and C (>50 µm). While only type A was detected in the case of WC-17 wt.% Co powder.

On the other hand, it has been reported that uniform and crack-free single tracks can be formed from nanostructured ball-milled powder with a composition of WC-75 wt.% Co [12]. However, another experimental approach showed that a decomposition of WC, cracking, and porosity in the Co-based matrix were predominant material characteristics when both high-energy milled and agglomerated and sintered nanostructured WC-12 wt.% Co powders have been processed [13]. Also, it has been observed that nano-sized and submicron WC particles have completely dissolved when a powder mixture had a Co content of 50 wt.%, and can only be retained with Co of just 6 wt.% [14]. Moreover, a mixture containing 50 wt.% WC has not been recommended for L-PBF processing due to the cracking tendency because of the formation of high fraction of brittle ternary carbide phase [15].

The feasibility of conducting L-PBF using a different ceramic powder with a cobalt based matrix for application as a cutting tool material has been examined by Davydova et. al. [16]. In this study metal coated B_4_C powders were produced using a patented chemical vapor deposition (CVD) coating process. A highly porous structure of 37% containing grains of boron carbide embedded in the cobalt-based matrix was obtained and new carbide phases due to chemical interaction during laser heating were also formed.

Overall, the literature to date shows that the formations of cracks and pores, as well as WC phase decomposition are still the dominant problems facing the processing of the WC-Co system by L-PBF. Therefore, the main purpose of the present study was to explore the feasibility of the manufacture, by L-PBF, of a WC-Co cutting tool material using a novel, and atypical, two-phase powder feedstock. In this work, a commercially available tungsten carbide-based ceramic powder with a spherical morphology and size range 45–125 µm was selected and Co (1–3 µm diameter) was incorporated via a novel satelliting process previously reported [17] in order to adhere the fine Co onto the surface of the larger carbide particles. The choice of tungsten carbide powder diameter was governed by the need to ensure that a high proportion of the fine Co adhered to the ceramic phase [18]. Furthermore, a spherical tungsten carbide grain morphology was preferred to the more commonly available prismatic, angular type to improve powder flowability.

## 2. Materials and Methods

### 2.1. Characterization of the Powder Feedstock Used in the Present Research

Commercially available powders, Spherotene^®^ and Co, were used to prepare a composite feedstock. Spherotene^®^ is a mixed tungsten carbide containing WC/W_2_C/WC_(1−x)_ phases which is manufactured in the form of spherical particles by a proprietary cold crucible electromagnetic levitation process (Technogenia Group, Saint-Jorioz, France). In this paper, for conciseness, the Spherotene^®^ is denoted by the symbol WC_M_. The composite feedstock was prepared with a nominal composition of WC_M_-Co (88–12) wt.% by using an innovative powder preparation method known as ‘satelliting’ [17]. The Spherotene^®^ powder has a nominal size range of (45–125) µm and the Co metal powder (ABSCO Limited, Suffolk, UK) has a nominal particle size of (1–3) µm.

**Figure 1:**
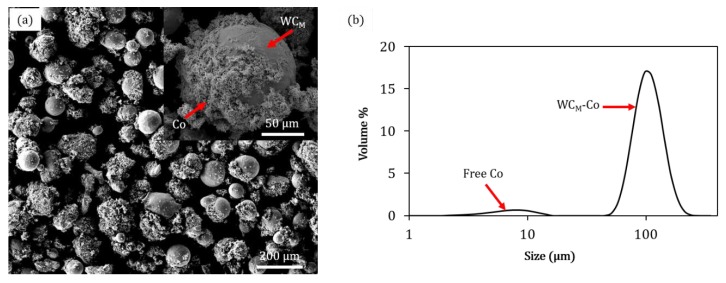
Scanning electron micrograph (SEM) of the satellited WC_M_-12 wt.% Co composite powder (**a**), and its size distribution obtained by laser diffractometry analysis after mixing by satelliting (**b**).

The mixing process was divided into two steps: the dry mixing step, where the WC_M_ and Co were mixed together with a certain weight ratio, and the wet step where poly-vinyl alcohol (PVA) binder was added in the ratio of 0.02 wt.% from the total weight of the batch. After that, the whole wet composite powder was mixed by a gyroscopic mixer for 20 min to complete the mixing process. The resultant wet composite powder was then distributed onto a tray and put in an oven for 12 h to dry, where the temperature was set to 50 °C. The resultant satellited composite powder is shown in Figure 1a, where the decoration of Co particles on a surface of a WC_M_ particle is presented in the upper right corner. It shows that the spherical shape is the main morphology of the WC_M_ particles. The satelliting process led to the production of a uniform distribution of WC_M_ particles where most of the Co particles are adhered to the WC_M_ particles’ surfaces. The size distribution for laser diffractometry analysis (Figure 1b) shows also that a small amount of Co phase is still free after satelliting.

### 2.2. Single-Track Formation during Laser Powder-Bed Fusion

A Realizer GmbH SLM-50, Germany, selective laser melting machine equipped with a continuous 100 W yttrium fibre laser (YLM-100-AC) was used to melt a single layer of satellited feedstock into tracks 5 mm long. To have consistent powder-bed thickness across experiments, a 304 stainless steel substrate was sand blasted prior to any melting to facilitate the spreading of the powders. The layer thickness was determined by referencing the substrate to the level of the machine platform. Then the substrate was lowered by 150 µm with respect to the machine platform. After that, the powder was spread on the substrate using the automatic re-coating system of the machine. Before the melting experiments, the laser was focused to a spot size of 22 µm (nominal spot size of the laser at full focus) at a full measured power of 100 W. The process parameters used to perform these single track experiments are listed in Table 1. The nominal scanning speed value was calculated from the ratio of point distance/exposure time. The point distance was fixed at 10 µm. For each process parameter set, three tracks were produced and characterized.

### 2.3. Characterization of the Single Tracks

The morphological measurements of the single tracks produced were taken at the middle segments to investigate the melting and solidification that occurs during the steady-state regime of laser. The width and height of these tracks was measured by an optical microscope (OM), Nikon Eclipse LV100ND. Care was taken to measure the width consistently at the track bottom/substrate boundary. The track height was calculated from the difference in vertical scale readings. The average track width and height was calculated from six measurements. The melt pools at the ends of tracks were defined by using scanning electron microscopy (SEM). It is noteworthy that the laser will decrease its acceleration of speed towards the end of the track to come to stop or a turn. However, in the present study it is assumed that the melt pools at the end of tracks are still representatives of the melt pool during steady state melting, at least in the first order of approximation. The length and width of the melt pool were calculated by fitting an ellipse around the solidification line of the last laser point exposure. The width was extracted from the minor axis considering the boundary between the solidified material and substrate.

For cross-sectional investigations, selected single tracks were cut perpendicular to the laser scanning direction using a SiC cutting disc and then mounted in conducting resin followed by the normal grinding/polishing procedure. The melted depth between the free surface of the substrate and the bottom of the molten pool as well as the length and width of melt pool morphology at the end of the track were measured from three representative images at each set of process parameters by using the open source software ImageJ 1.46r. For morphological analyses, an FEI Quanta600 (SEM) equipped with energy-dispersive X-ray analysis (EDXA) was used with 20 kV as an accelerating voltage. To differentiate WC_M_ particles sintered to the track to melt pool instabilities such as balling, backscattered (BSE) imaging was undertaken to differentiate between phases.

## 3. Results

### 3.1. Evolution of the Track Morphology as a Function of Laser-Scanning Speed

**Figure 2:**
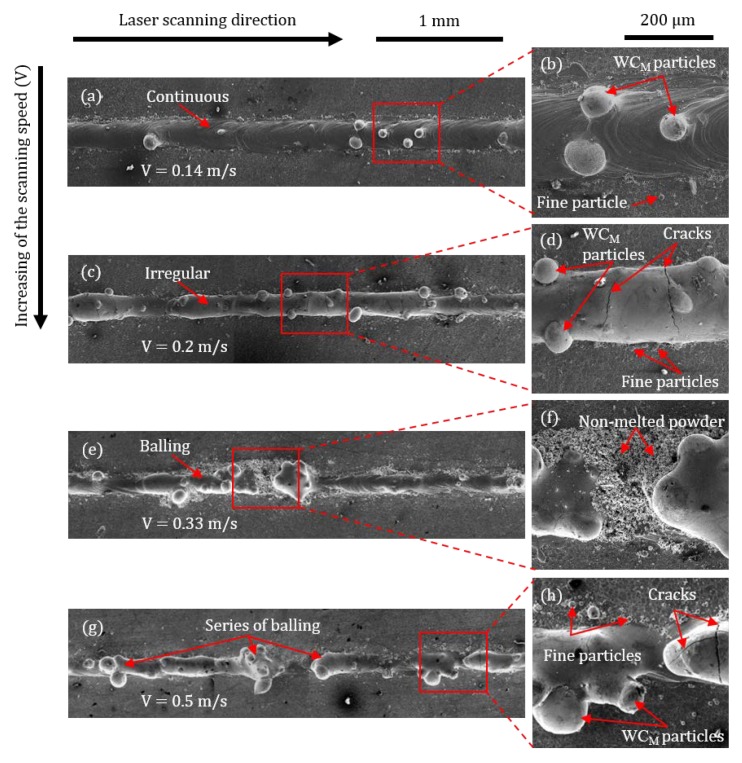
Consolidation behaviours of single tracks at scanning speeds of 0.14 m/s (**a**), 0.2 m/s (**c**), 0.33 m/s (**e**), and 0.5 m/s (**g**), with corresponding higher magnifications track images in (**b**), (**d**), (**f**) and (**h**), respectively.

The morphology of the four tracks differ vastly with an increase in laser-scanning speed. At a lower scanning speed of 0.14 m/s (Figure 2a), a continuous track with no visible cracks and relatively uniform surface morphology can be observed. Images at higher magnification (Figure 2b) indicate two more aspects; WC_M_ particles appear on the top surface of the track and relatively fine particles are also observed on the substrate near the track sides. With an increase of the scanning speed to the value of 0.2 m/s, the track surface morphology becomes more irregular (Figure 2c). Cracks can clearly be observed on the outside track surface at a higher magnification (Figure 2d). Balling is significant at a scanning speed of 0.33 m/s: this leads to numerous discontinuities where the solidified material is accumulated in some places and diminished in others (Figure 2e). Moreover, non-melted powder clearly appears at higher magnification (Figure 2f). An increase in the scanning speed to 0.5 m/s has led to extensive discontinuities and balling, as shown in Figure 2g. In addition, the higher magnification image shows that the formation of fine particles along the track sides and cracks becomes predominant, as presented in Figure 2h.

### 3.2. Track Width, Height, and Melt-Pool Length Measurements

**Figure 3:**
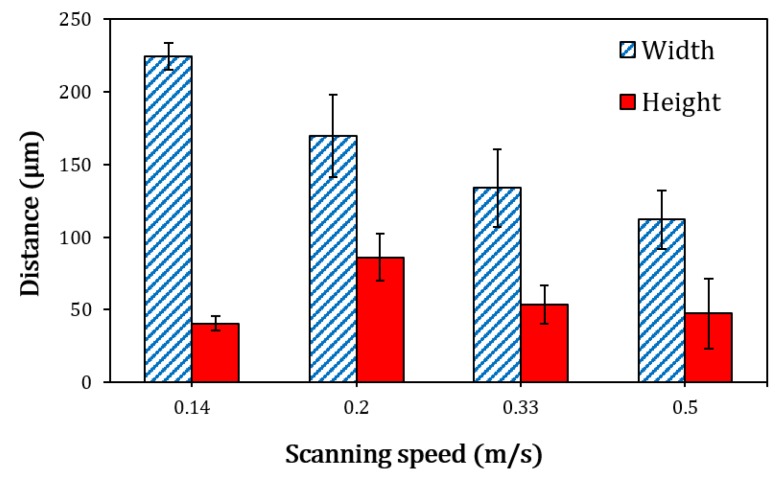
Variation in track widths and heights with respect to the laser scanning speed. Noting track height does not vary significantly with the parameters evaluated here.

Figure 3 shows the variation of track width and height as a function of laser-scanning speed. It is noticed that the track reached a maximum width of 225 ± 10 µm at a scanning speed of 0.14 m/s. The track width decreases to a minimum of 112 ± 20 µm when the laser-scanning speed is increased to 0.5 m/s. It was observed that the track width decreases consistently when the scanning speed increases although precise measurements are challenging due to the irregular morphology of the tracks. On the other hand, the track height does not follow a regular trend within the explored parameter space. It increases from 40 ± 5 µm to 86 ± 16 µm when the scanning speed increases from 0.14 m/s to 0.2 m/s, while further increase of the scanning speed to 0.5 m/s leads to a decrease in track height to 47 ± 24 µm where highest uncertainty value can be observed indicating track morphology irregularities.

**Figure 4:**
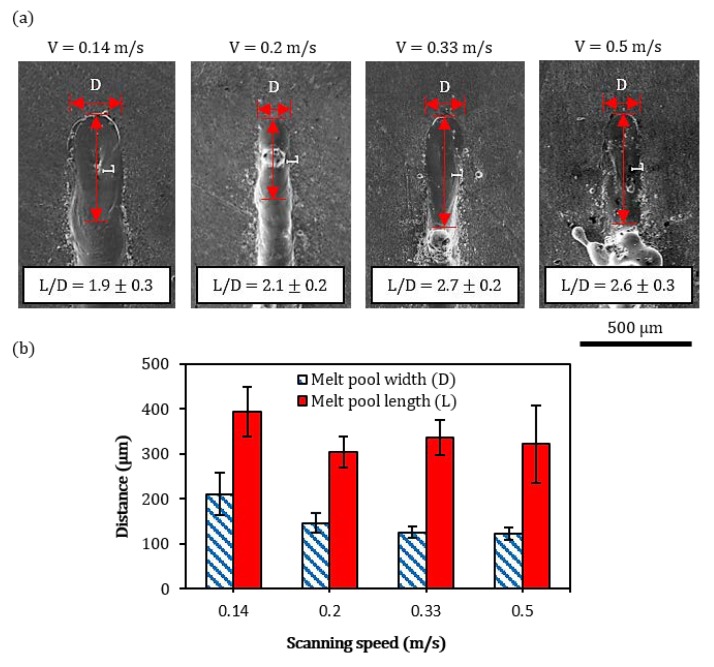
(**a**) Plan view images of representative melt pool shapes at the end of the solidified tracks, and (**b**) bar chart showing the measurements of the corresponding melt pool widths and lengths.

The effect of laser-scanning speed on the evolution of melt pool shapes at the ends of the solidified tracks is presented in Figure 4a. The results confirm the previous trend, where the reduction in melt pool width can be observed as the scanning speed increases. It shows that the ratio of melt-pool length (L), which represents the major axis of the first elliptical edge that appeared on the surface far from the melt pool centre, to its width (D), increases from 1.9 ± 0.3 to 2.6 ± 0.3 as the scanning speed increases from 0.14 m/s to 0.5 m/s. The melt pool width decreases from 210 ± 47 µm to 122 ± 14 µm when the scanning speed increases from 0.14 m/s to 0.5 m/s as shown in Figure 4b. No significant variations between the melt pool width and the measured track width are observed confirming that the method of investigation produces accurate estimates of the melt pool produced during the steady-state laser melting. No noticeable variations in melt pool length are observed as a function of laser-scanning speed.

### 3.3. Cross-Sections and Melted-Depth Characteristics

**Figure 5:**
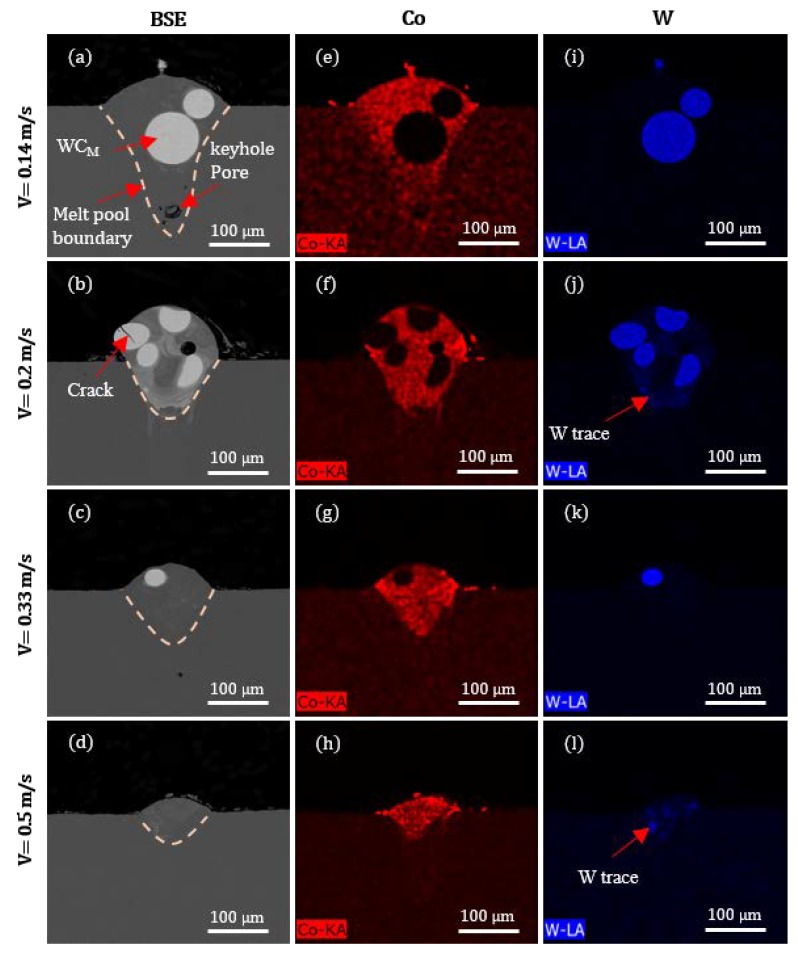
Backscattered (BSE) images and energy-dispersive X-ray (EDX) composition maps obtained from cross-sections of melt tracks for different laser scanning speeds. (**a**–**d**) BSE images for scanning speeds of 0.14, 0.2, 0.33 and 0.5 m/s respectively. (**e**–**h**) EDX maps for Co at scanning speeds of 0.14, 0.2, 0.33, and 0.5 m/s, respectively. (**i**–**l**) EDX maps for W at scanning speeds of 0.14, 0.2, 0.33 and 0.5 m/s respectively.

The cross-sectional investigations of the tracks are presented in Figure 5. BSE imaging (Figure 5a–d) reveals that WC_M_ particles are present inside the metal matrix, although partial dissolution may have taken place. Keyhole porosity is occasionally observed as shown in Figure 5a. The limits of the melt pools are defined from the Co element energy-dispersive X-ray (EDX) maps (Figure 5e–h). The melt pool geometry becomes shallow and narrow as the laser scanning speed is increased. A progression of a crack from the outer solidified surface for a specific distance inside a WC_M_ particle through its centre appears at a scanning speed of 0.2 m/s (Figure 5b). For relatively low applied energies (corresponding to relatively higher scanning speeds), it can be observed that there is a reduction of the dissolution of the WC_M_ particles with only traces of W appearing in the melt pool at scanning speeds of 0.2 m/s to 0.5 m/s as shown in Figure 5j,l, respectively.

**Figure 6:**
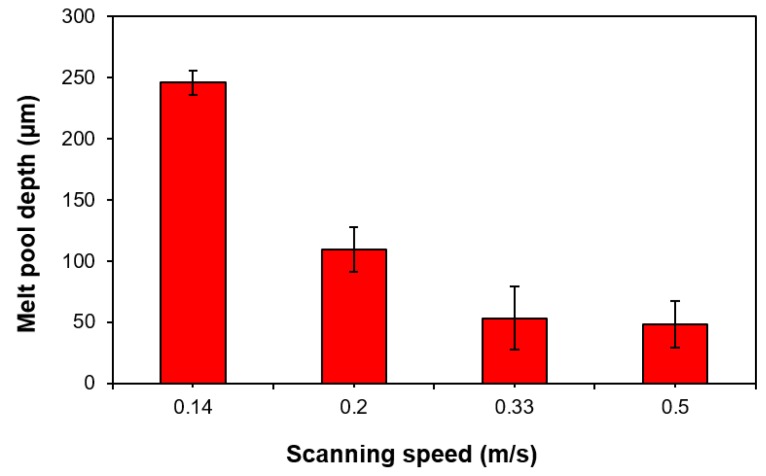
Bar chart showing the effect of laser-scanning speed on melt-pool depth.

The variation of melted depth as a function of scanning speed value is illustrated in Figure 6. This shows that the increase of the scanning speed from 0.14 m/s to 0.5 m/s leads to a decrease of the melted depth from 245 ± 10 to 48 ± 20 µm. Moreover, it can be observed that the measured uncertainties are proportional to the increase of scanning speed. The morphological irregularities observed for scanning speed >0.14 m/s are also reflected in the substantial variations of the measured melt pool depth along the tracks.

## 4. Discussion

### 4.1. The Evolution of the Track Morphologies as a Function of Laser-Scanning Speed

The objective of this study was to produce a uniform track morphology free from cracking and porosity. This presented several challenges due to the large particle-size distribution of the feedstock. In the first place, the physical size of the individual particles affected significantly the material melting during laser processing. It is well known that when the laser scans over the applied powder bed, energy is absorbed by the surface of individual particles [19]. This leads to an increase of the temperature of particle surface before heat can penetrate to the centre of powder. By taking into consideration the thermal conductivity and melting point of WC_M_ (84 W/m/K and 2687 °C), Co (100 W/m/K and 1495 °C), and steel (68 W/m/K and 1435 °C), respectively [20], the location of the Co fine particles (which effectively decorate WC_M_), and the difference in physical size, it is likely that Co powder and the surface of steel substrate started to melt first to form the molten pool. Molten Co then reacted with larger WC_M_ particles as demonstrated by the partial undesirable dissolution of WC_M_ observed in the tracks for lower scanning speeds (Figure 5j,l).

In addition, the large particle size distribution imposed the necessity to use an unconventional layer thickness of 150 µm (most of the L-PBF equipment is typically using a layer thickness of 30–50 µm). This, combined with the low thermal conductivity of the feedstock and a laser spot size of 22 µm, required a relatively long laser dwelling time to accommodate and homogenize the temperature of Co within a given layer thickness. A common way to describe the combined effect of process parameters on the material densification during L-PBF processing is expressed by the volumetric energy density (VED) as given in Equation (1) [21]:

VED = P/vdt (J/mm^3^) (1)

where P is the laser power (W), v, d, and t are the laser-scanning speed (mm/s), laser beam diameter (mm), and applied layer thickness (mm), respectively. In this study the applied VED ranged between 60 and 215 J/mm^3^—figures comparable or higher to the processing of other structural alloys in the same L-PBF platform. A comparison with the literature of additive manufactured WC composites is instead non-trivial as energy densities are often calculated using hatch distance (distance between two adjacent tracks) while the spot size is not reported [9,10].

**Figure 7:**
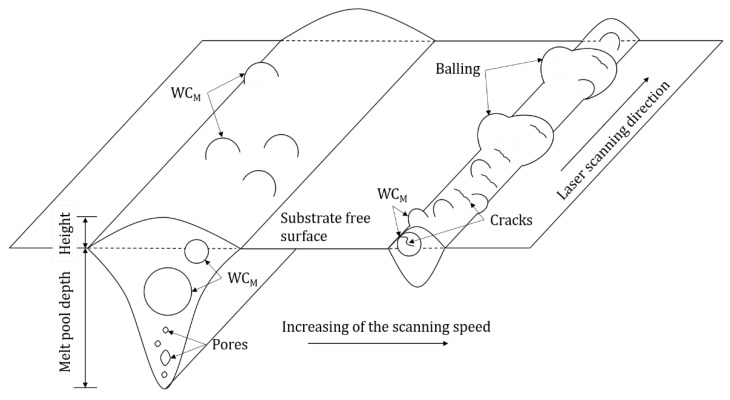
A schematic representation of the effect of scanning speed on melt pool shape showing a transition in consolidation regimes.

A schematic representation of the effect of scanning speed on the evolution of melt pool geometry from current results is shown in Figure 7. Within the range of experimental parameters investigated, it was noted that the width and the depth of the tracks varies significantly with the laser-scanning speed (widest tracks and highest penetration depths are observed for lowest laser-scanning speeds), while no definite trends can be evinced regarding the track height.

Particular attention was dedicated to the analysis of the material accumulation along the track sides—in the present investigation in the form of balling and sintered particles—as this can impose severe barriers to the fabrication of successful components in 3D. The presence of fine particles can be non-melted power or spatters which were sintered on/near the track surface without melting due to their existence in the heat-affected zone [22].

With the exception of the tracks produced at laser scanning speed of 0.14 m/s, extensive balling was observed along the track edges. Balling is a consequence of capillary instability of the melt pool [21]. According to the Rayleigh–Plateau criteria, balling can be modelled as a cylindrical volume of liquid, with a length (*l*) and a diameter (*d*), on the substrate. The instability condition of such a free cylinder is satisfied when its harmonic disturbances have wavelengths comparable to *l*, or *l*/*d* > π. Under this condition, the cylinder minimizes its surface energy by breaking into droplets. As observed in Figure 4, the increase of laser-scanning speed produced higher values of the L/D elongation ratio and, therefore, supported the break-up of the melt pool into large spherical deposits.

Sintered WC_M_ particles were also occasionally found outside the tracks surfaces. From a practical point of view, sintered WC_M_ particles have the same deleterious influence associated to balling as they limit the spreading of powders in successive layers and might encourage melt pool break-up during the deposition of continuous tracks. The analysis of the tracks suggested that fewer substrate interactions due to increasing the scanning-speed values leads, at least qualitatively, to higher sensitivity to cracking (Figure 2d,h). An increase in the laser scanning speed might lead to relatively higher cooling rates and temperature gradients resulting in high residual thermal stresses [23].

Finally, it is noteworthy that the track height for the most uniform track morphology produced at a scanning speed of 0.14 m/s was 40 ± 5 µm, as shown in Figure 3, which gives a ratio of ~0.27 with respect to the applied layer thickness of 150 µm. This indicates a low packing density of the feedstock, as compared to a ratio up to 0.8 for 316 L stainless steel powder [21], which can affect the bulk density when a multi-layered component wanted to be fabricated.

### 4.2. Melting Regimes as a Function of Laser-Scanning Speed

**Figure 8:**
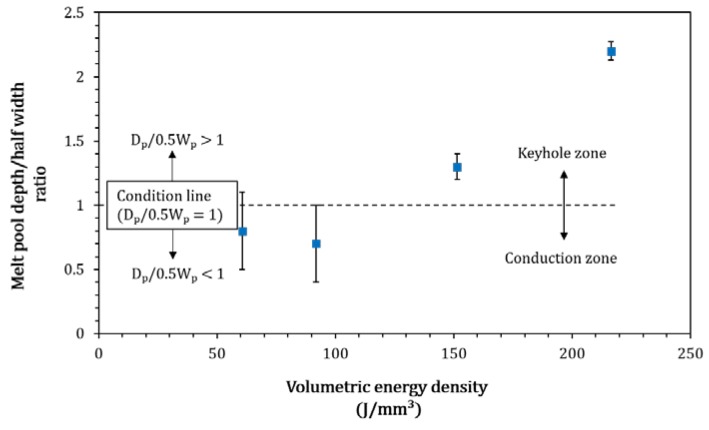
The ratio of melted depth/half of track width as a function of volumetric energy density, with maximum and minimum limits for specimens created in this study. The variation in measurements is consistent with instability during the consolidation process.

The substrate penetration depth depends strongly on the laser scanning speed as shown in Figure 6. It is useful to classify the melting behaviour observed in the tracks according to two melting regimes defined in the literature: conduction mode melting, for which the penetration depth (D_p_) is approximately equal to half of melt pool width (W_p_) (e.g. D_p_/0.5W_p_ ≤ 1), and keyhole mode melting for which D_p_/0.5W_p_ > 1 [24]. As shown in Figure 8, for high values of VED (>150 J/mm^3^) keyhole mode melting is observed. Interestingly, this is a suitable condition for the production of continuous tracks.

This research has revealed several challenges related to the laser melting of a feedstock exhibiting a large particle-size distribution. Firstly, in order to melt a relative thick powder bed, long laser exposures are required. In the present investigation, continuous tracks are only formed by reaching a key-hole mode melting regime, where porosity and melt pool dynamics are, however, difficult to predict and control. A possible solution to minimise key-holing might be that of using a laser of higher power and larger spot size to directly increase the melt pool width. Secondly, during the laser exposure, regardless of the parameters used, given the marked particle size difference, the fine Co particles are likely to melt first with only partial dissolution of WC_M_. Although the preservation of WC_M_ might be beneficial from a microstructural point of view, large WC_M_ are impractical for successful spreading of subsequent layers of the powders and homogenous melting. The positive effect of a ductile substrate, which forms a ductile matrix resistant to crack formation for most of the processing conditions explored in this investigation, is notable.

## 5. Conclusions

In this study for the first time, the satelliting method was used to prepare a WC_M_-12 wt.% Co composite powder for L-PBF processing. The results showed that the feedstock is highly sensitive to laser-scanning speed and only a small processing window was capable to produce continuous tracks. It was found that:

- For VED > 200 J/mm^3^, continuous tracks of WC_M_-12 wt.% Co can be formed; the tracks present minimal cracking and porosity but large WC_M_ particles are found to be sintered to sides of the tracks.
- For VED < 200 J/mm^3^, as the energy is decreased, tracks become discontinuous with evidence of balling and powder bed denudation.
- A clear transition between conduction to keyhole-mode melting with respect to the values of volumetric energy density was observed and correlated to the melt-pool characteristics.
- The low packing density of the feedstock produced significant shrinkage during melting; this should be taken into account when a multi-layered component is to be fabricated.

## Figures and Tables

**Table 1:** The process parameters used to produce the single tracks investigated in this study.

Process Parameters	Values (units)/Direction			
Measured powder layer thickness	150 (µm)			
Oxygen level	<0.5%			
Substrate pre-heating temperature	200 (°C)			
Scanning strategy	Uni-directional			
Measured laser power	100 (W)			
Exposure time	70 (µs)	50 (µs)	30 (µs)	20 (µs)
Nominal laser scanning speed	0.14 (m/s)	0.2 (m/s)	0.33 (m/s)	0.5 (m/s)
